# The effect of exposure to biomass smoke on respiratory symptoms in adult rural and urban Nepalese populations

**DOI:** 10.1186/1476-069X-13-92

**Published:** 2014-11-06

**Authors:** Om P Kurmi, Sean Semple, Graham S Devereux, Santosh Gaihre, Kin Bong Hubert Lam, Steven Sadhra, Markus FC Steiner, Padam Simkhada, William CS Smith, Jon G Ayres

**Affiliations:** Clinical Trials Service Unit & Epidemiological Studies Unit, Nuffield Department of Population Health, University of Oxford, Oxford, OX3 7LF UK; Scottish Centre for Indoor Air, Division of Applied Health Sciences, School of Medicine, University of Aberdeen, Aberdeen, AB24 3FX UK; Department of Child Health, Royal Aberdeen Children’s Hospital, University of Aberdeen, Aberdeen, AB25 2ZG UK; Institute of Occupational and Environmental Medicine, University of Birmingham, Birmingham, B15 2TT UK; School of Health and Related Research, University of Sheffield, Sheffield, S10 2TN UK; School of Medicine and Dentistry, University of Aberdeen, Aberdeen, AB24 3FX UK

**Keywords:** Respiratory symptoms, Breathlessness, Phlegm, Solid fuel, Household air pollution

## Abstract

**Background:**

Half of the world’s population is exposed to household air pollution from biomass burning. This study aimed to assess the relationship between respiratory symptoms and biomass smoke exposure in rural and urban Nepal.

**Methods:**

A cross-sectional study of adults (16+ years) in a rural population (n = 846) exposed to biomass smoke and a non-exposed urban population (n = 802) in Nepal. A validated questionnaire was used along with measures of indoor air quality (PM_2.5_ and CO) and outdoor PM_2.5_.

**Results:**

Both men and women exposed to biomass smoke reported more respiratory symptoms compared to those exposed to clean fuel. Women exposed to biomass were more likely to complain of ever wheeze (32.0 % vs. 23.5%; p = 0.004) and breathlessness (17.8% vs. 12.0%, p = 0.017) compared to males with tobacco smoking being a major risk factor. Chronic cough was similar in both the biomass and non-biomass smoke exposed groups whereas chronic phlegm was reported less frequently by participants exposed to biomass smoke. Higher PM_2.5_ levels (≥2 SDs of the 24-hour mean) were associated with breathlessness (OR = 2.10, 95% CI 1.47, 2.99) and wheeze (1.76, 1.37, 2.26).

**Conclusions:**

The study suggests that while those exposed to biomass smoke had higher prevalence of respiratory symptoms, urban dwellers (who were exposed to higher ambient air pollution) were more at risk of having productive cough.

**Electronic supplementary material:**

The online version of this article (doi:10.1186/1476-069X-13-92) contains supplementary material, which is available to authorized users.

## Introduction

While the major cause of respiratory health problems among adults in the developed world is smoking, exposure to particles generated from biomass smoke is a major cause of respiratory diseases in low income countries [1, 2].

Several studies have reported higher prevalence of respiratory ill-health among adults exposed to biomass smoke with estimated risk ratios between 1.2 and 7.9 [3]. Most studies showing an association between household air pollution and respiratory health problems used proxy measurements of exposure such as the number of hours spent on cooking, or simply ever used particular fuels. In addition, not all of the important confounders, particularly socio-economic status, smoking, fuel types and age, have been adjusted for in earlier studies. Although positive associations between chronic bronchitis and household air pollution have been reported, there were large variations in the prevalence of different respiratory symptoms [4, 5]. A meta-analysis [3] reported positive associations between the use of solid fuels and both chronic obstructive pulmonary disease (COPD) (OR = 2.80, 95% CI 1.85, 4.00) and chronic bronchitis (OR = 2.32, 95% CI 1.92, 2.80) but also highlighted considerable heterogeneity in design, measurement, and sizes of effect estimates in studies from different low and middle income countries. To date, only one study from Nepal has reported a relationship between directly measured household exposure and respiratory symptoms although the exposure assessment was carried out only during cooking [6].

This study aimed to investigate the relationship between respiratory symptoms and lung function and direct measures of exposure to emissions from biomass and non-biomass (particularly liquefied petroleum gas [LPG]) fuels in rural and urban households in Nepal. We previously reported a 20% prevalence of COPD in the biomass exposed population compared to 11% in the urban population based on spirometry data [7]. In this report we focus on the respiratory symptoms.

## Methods

### Design and study sample

This cross-sectional study was carried out between April 2006 and February 2007. The biomass-exposed population (98.9% used wood) was sampled from two village development committees (VDCs) in the Kathmandu Valley. Four wards (out of nine) in each VDC were randomly selected and all individuals in the selected wards aged ≥16 years were eligible if they met the inclusion criteria (no doctor diagnosed major respiratory or cardiovascular health problems and agreement to 24-h continuous airborne exposure monitoring in their homes). The non-exposed population (98.4% used LPG) were selected from six wards (from a total of 35) in the Kathmandu municipality: three were selected randomly near the ring road and the other three selected from 1–2 km inside the ring road. Further details were published elsewhere [7, 8]. The sample size was based on lung function assuming a prevalence of COPD of 10% in the non-exposed and 20% in the exposed populations, the latter being twice the reported prevalence for Nepalese populations [7].

An interviewer-administered questionnaire was used to collect data on smoking, socio-economic status, literacy, kitchen characteristics, cooking details, history of fuel use, and respiratory symptoms. The questionnaire was translated into Nepalese and back translated into English by an independent translator and a pilot study was conducted to identify issues of logistics and understanding. The study protocol was approved by the Nepal Health Research Council. Written, informed consent was obtained from all study participants.

### Measurements of exposure

Levels of particulate matter with an aerodynamic diameter <2.5 μm (PM_2.5_) were measured over a continuous 24-h period in most dwellings (n = 490) using photometric devices (SidePak AM510 and DustTrak Model 8520, TSI Inc., Shoreview, MN, USA), from which mean 24-h PM_2.5_ (in μg/m^3^) was derived. We report here the results from 442 households (206 biomass burning; 236 non-biomass burning) which had at least 20 h data. Outdoor PM_2.5_ concentrations were measured in both rural and urban areas on the veranda (for logistic and security reasons) in 118 homes (46 biomass burning, 72 non-biomass burning). Indoor 24-h carbon monoxide (CO) concentrations (in ppm) were measured in 126 homes (40 biomass burning, 86 non-biomass burning) using HOBO CO loggers (MicroDAQ, Contoocook, NH, USA). The direct reading photometric instruments were calibrated using data from co-located gravimetric samplers [8].

### Assessment of respiratory outcomes

Respiratory symptoms were based on the Medical Research Council (MRC) questionnaire and included cough, phlegm, breathlessness, and wheezing/whistling. Breathlessness was measured using the five-level modified MRC (mMRC) dyspnoea scale [9]. In this report we define breathlessness as those falling into Grade 2 or above. Participants who reported to have cough or bring up phlegm first thing in the morning for at least three months each year were considered to have chronic cough or phlegm, respectively. Chronic bronchitis was defined as the presence of both chronic cough and chronic phlegm. Participants also underwent spirometry as reported elsewhere [9]. For comparability with previous studies, we defined airflow obstruction in two ways: (i) forced expiratory volume in 1 s (FEV_1_) to forced vital capacity (FVC) ratio less than the lower limit of normal (LLN); or (ii) FEV_1_/FVC <0.70 [7].

### Covariates

Height and weight were measured using standard protocols [10], from which body mass index (BMI) was computed (in kg/m^2^). Participants were classified as non-, ex- and current smokers, where the latter two categories had smoked at least 20 packs of cigarettes or 360 g of tobacco in a lifetime, or at least one cigarette per day or one cigar a week for one year. We also collected information on exposure to environmental tobacco smoke and current occupation. We used monthly household income (in Nepalese Rupees where 1 US$ ≈ 100 Rs) and educational level as proxies for socio-economic status.

### Statistical analysis

Statistical analyses were performed using STATA (version 12, College Station, TX, USA). Results for PM_2.5_ and CO concentrations are expressed as geometric means and geometric standard deviations unless indicated otherwise. Mean indoor PM_2.5_ concentrations >2 standard deviations (SD) of the arithmetic means over the entire sampling window were also calculated from the real time exposure data and are reported here to assess any associations between dependent variables and maximal exposures (i.e. during cooking). Baseline demographic characteristics were compared between biomass and non-biomass exposed participants separately for men and women by regression taking into account the household clustering effect. Regression models were constructed to evaluate the effect of pollutants (biomass, exposure to PM_2.5_ and CO independently) on respiratory symptoms. All known and potential confounders (age, income, educational level, smoking status, and BMI) were adjusted for to obtain regression coefficients (β) with robust variance estimates to allow for household clustering.

## Results

Among 1648 participants (762 men and 886 women) enrolled, 846 (51%) used biomass and 802 (49%) used non-biomass fuels (primarily LPG), respectively. The proportion of current smokers, underweight, illiterate and those having lower income were higher among biomass users (Table 1). Around 35% of rural women had smoked at some point in their lives compared to only 9% of urban dwellers.

**Table 1:** **Demographic data of 1648 Nepalese adult men and women according to household fuel type**

	Men			Women		
	Biomass	Non-biomass	p	Biomass	Non-biomass	p
n	382	380		463	423	
Age (years); mean (SD)	35.7 (17.1)	35.2 (15.1)	0.612	36.1 (17.1)	34.5 (15.1)	0.076
Height (cm); mean (SD)	162.3 (7.4)	166.1 (6.8)	<0.001	149.8 (5.8)	153.0 (6.2)	<0.001
Weight (kg); mean (SD)	52.6 (8.2)	61.9 (10.2)	<0.001	46.2 (7.2)	56.0 (10.3)	<0.001
Body mass index (kg/m^2^); n (%)						
<18.5	106 (27.8)	58 (15.3)	<0.001	111 (24.0)	38 (9.0)	<0.001
18.5–24.99	258 (67.5)	233 (61.3)		314 (67.8)	228 (53.9)	
≥25	18 (4.7)	89 (23.4)		38 (8.2)	157 (37.1)	
Educational level; n (%)						
Undergraduate or higher	16 (4.2)	173 (45.5)	<0.001	3 (0.7)	95 (22.5)	<0.001
Up to 12 years of formal education	51 (13.4)	83 (21.8)		24 (5.2)	94 (22.2)	
Up to 10 years of formal education	76 (19.9)	54 (14.2)		42 (9.1)	56 (13.2)	
<10 years of formal education	174 (45.6)	60 (15.8)		143 (30.9)	94 (22.2)	
Illiterate	65 (17.0)	10 (2.6)		251 (54.2)	84 (19.9)	
Farmer; n (%)	357 (93.7)	59 (15.5)	<0.001	441 (95.7)	81 (19.2)	<0.001
Monthly household income (Rs*); mean (SD)	6173 (8486)	21782 (19143)	<0.001	6452 (8417)	22977 (22839)	<0.001
Smoking status; n (%)						
Non-smoker	210 (55.0)	247 (65.0)	<0.001	299 (64.6)	385 (91.0)	<0.001
Ex-smoker	30 (7.9)	57 (15.0)		51 (11.0)	23 (5.4)	
Current smoker	142 (37.2)	76 (20.0)		113 (24.4)	15 (3.6)	
Age started smoking (years); n (%)						
<11	28 (16.3)	10 (7.5)	<0.001	46 (28.0)	8 (21.1)	0.792
11-15	47 (27.3)	8 (6.0)		28 (17.1)	8 (21.1)	
16-20	50 (29.1)	53 (39.8)		53 (32.3)	12 (31.6)	
>20	47 (27.3)	62 (46.6)		37 (22.6)	10 (26.3)	
Environmental tobacco smoke exposure; n (%)	291 (76.2)	194 (51.1)	<0.001	354 (76.5)	167 (39.5)	<0.001
≥10 years of current fuel use; n (%)	317 (83.0)	173 (45.5)	<0.001	393 (84.9)	187 (44.2)	<0.001

*Nepalese Rupees (1 US$ ≈ 100 Rs).

The geometric mean (± geometric SD) 24-h indoor PM_2.5_ concentration in biomass using homes was significantly greater than in non-biomass using homes (455 ± 2.4 vs. 101 ± 2.0 μg/m^3^, p <0.001), although there was no significant difference in outdoor air pollution between biomass and non-biomass using homes (129 ± 2.7 vs. 115 ± 2.5 μg/m^3^, p = 0.249). PM_2.5_ measured concurrently on the veranda and 100 m from five biomass burning houses showed substantially higher concentrations (129 ± 1.5 μg/m^3^) compared to the outdoor environment (7.4 ± 2.8 μg/m^3^). Mean peak indoor PM_2.5_ (defined as >2 SD of the mean level over the entire sampling window) was 1790 μg/m^3^ in homes using biomass and 141 μg/m^3^ in non-biomass homes (arithmetic means 2828 and 335 μg/m^3^, respectively).

The 24-h CO concentrations in kitchens using biomass fuel were significantly higher than in non-biomass fuel homes (13.4 ± 2.2 vs. 2.0 ± 2.0 ppm, p <0.001). The levels of PM_2.5_ and CO were much higher during cooking particularly in those houses where biomass was used as cooking fuel (Additional file 1: Figure S1).

In general symptom prevalence increased with age (Additional file 2: Table S1). Table 2 presents age-adjusted prevalence of breathlessness, wheeze, and chronic bronchitic symptoms. Dsypnoea (mMRC ≥ Grade 2) and wheezing (ever or on most days/nights) were more common among biomass users compared to those who used non-biomass fuel (p <0.001), with age-adjusted prevalence of dyspnoea being 17.8% (95% CI 14.1, 21.5%) among female and 12.0% (8.9, 15.1%) among male biomass fuel users, compared to 7.6% (5.1, 10.1%) and 2.5% (1.0, 4.1%) among cleaner fuel users. Likewise, wheezy chest was approximately three times more likely to be reported by those using biomass fuel. On the other hand, male non-biomass users reported significantly more chronic phlegm (12.9%; 95% CI 9.6, 16.3%) compared to biomass users (3.0%; 1.4, 4.7%, p <0.001). Such difference was not observed in females. When restricting to non-smokers, all symptom prevalence was lower, although not statistically different from the entire sample. Dyspnoea and wheeze were more prevalent among biomass users. In contrast the prevalence of chronic phlegm was higher in non-biomass users (p <0.001), both in males and in females (p = 0.041).

**Table 2:** Respiratory symptoms* in Nepalese adult men and women according to household fuel type

	Men					Women				
	Biomass		Non-biomass			Biomass		Non-biomass		
	n	% (95% CI)	n	% (95% CI)	p	n	% (95% CI)	n	% (95% CI)	p
	382		380			463		423		
mMRC scale ≥ Grade 2	48	12.0 (8.9, 15.1)	9	2.5 (1.0, 4.1)	<0.001	86	17.8 (14.1, 21.5)	30	7.6 (5.1, 10.1)	<0.001
Wheeze										
Ever	91	23.5 (19.5, 27.4)	32	8.7 (5.6, 11.7)	<0.001	152	32.0 (28.0, 36.0)	41	10.3 (7.4, 13.2)	<0.001
On most days/nights	71	18.2 (14.5, 21.9)	22	6.0 (3.4, 8.6)	<0.001	122	25.7 (21.8, 29.6)	30	7.5 (5.0, 9.9)	<0.001
Chronic cough	19	4.7 (2.7, 6.7)	19	5.3 (3.0, 7.6)	0.719	22	4.4 (2.5, 6.3)	16	4.2 (2.3, 6.0)	0.864
Chronic phlegm	12	3.0 (1.4, 4.7)	48	12.9 (9.6, 16.3)	<0.001	19	4.0 (2.0, 6.0)	24	5.8 (3.6, 8.0)	0.223
Chronic cough and phlegm	9	2.2 (0.8, 3.7)	13	3.6 (1.6, 5.6)	0.292	13	2.6 (1.2, 4.1)	9	2.3 (0.9, 3.7)	0.753
Non-smokers only	210		247			299		385		
mMRC scale ≥ Grade 2	16	8.6 (4.7, 12.5)	3	1.1 (−0.1, 2.3)	<0.001	37	13.8 (9.8, 17.9)	20	4.7 (2.7, 6.8)	<0.001
Wheeze										
Ever	31	15.3 (10.1, 20.4)	16	6.3 (3.3, 9.3)	0.004	66	23.8 (19.1, 28.6)	27	6.5 (4.1, 9.0)	<0.001
On most days/nights	22	10.9 (6.4, 15.4)	11	4.3 (1.8, 6.8)	0.014	58	20.9 (16.4, 25.5)	22	5.3 (3.2, 7.5)	<0.001
Chronic cough	8	4.3 (1.5, 7.2)	7	2.6 (0.7, 4.4)	0.305	6	2.3 (0.3, 4.4)	9	2.1 (0.8, 3.4)	0.867
Chronic phlegm	5	2.7 (0.4, 5.0)	18	6.7 (3.7, 9.7)	0.041	6	2.0 (0.4, 3.6)	19	4.9 (2.7, 7.2)	0.041
Chronic cough and phlegm	5	2.8 (0.4, 5.2)	3	1.1 (−0.2, 2.4)	0.241	4	1.5 (0.0, 2.9)	6	1.5 (0.3, 2.6)	0.989

*Adjusted for age.

Adjusting for potential confounders, those using biomass were associated with a significantly increased risk in breathlessness and wheeze. The increase in risk for dyspnoea was larger among men (OR = 7.88; 95% CI 2.84, 21.88) than among women (3.90; 2.00, 7.79), but the opposite was true for wheeze (Table 3). There was a negative association between biomass use and chronic phlegm prevalence, although this was significant only in men (OR = 0.21; 95% CI 0.09, 0.47). The magnitude of risk estimate was smaller when other measures of indoor pollutants (24-h mean PM_2.5_, PM_2.5_ >2 SD and CO) were used. Whilst the level of PM_2.5_ was much lower outdoors compared to indoors, there was a positive relationship between outdoor PM_2.5_ and chronic phlegm in both sexes, although neither reached statistical significance. Restriction to non-smokers made no material changes in the risk estimates, with statistical significance disappeared in wheeze among males due to the reduction in power (data not shown).

**Table 3:** Adjusted* odds ratios for respiratory symptoms and airflow obstruction according to exposure to particulate pollution

	Biomass		Indoor 24-h mean PM _2.5_		PM _2.5_ > 2SD of 24-h mean		Outdoor 24-h mean PM _2.5_		Indoor 24-h mean CO	
	OR (95% CI)	p	OR ^†^ (95% CI)	p	OR ^†^ (95% CI)	p	OR ^†^ (95% CI)	p	OR ^†^ (95% CI)	p
**Men**										
**Breathlessness**										
mMRC scale ≥ Grade 2	7.88 (2.84, 21.88)	<0.001	3.10 (1.53, 6.31)	0.002	2.67 (1.50, 4.78)	0.001	1.24 (0.16, 9.63)	0.837	8.33 (0.74, 93.27)	0.085
**Wheeze/whistling**										
Ever	2.48 (1.28, 4.82)	0.007	1.61 (0.96, 2.69)	0.070	1.72 (1.20, 2.46)	0.003	3.06 (1.28, 7.31)	0.012	1.28 (0.43, 3.83)	0.656
On most days and nights	2.03 (0.99, 4.16)	0.053	1.20 (0.65, 2.19)	0.563	1.29 (0.87, 1.91)	0.202	3.31 (1.35, 8.13)	0.009	1.35 (0.34, 5.31)	0.666
**Cough/phlegm**										
Chronic cough	0.87 (0.28, 2.69)	0.803	1.45 (0.68, 3.12)	0.339	1.28 (0.78, 2.09)	0.331	9.68 (0.59, 159.2)	0.112	0.73 (0.12, 4.52)	0.739
Chronic phlegm	0.21 (0.09, 0.47)	<0.001	0.69 (0.36, 1.33)	0.268	0.66 (0.43, 1.01)	0.055	2.68 (0.83, 8.67)	0.100	0.56 (0.13, 2.41)	0.432
Chronic bronchitis	0.62 (0.17, 2.31)	0.476	1.75 (0.70, 4.39)	0.233	1.09 (0.60, 1.97)	0.773	3.02 (0.32, 28.02)	0.331	Not estimable	-
**Airflow obstruction**										
FEV_1_/FVC < 0.70	1.94 (1.05, 3.59)	0.035	0.81 (0.46, 1.45)	0.480	1.24 (0.83, 1.86)	0.297	2.24 (0.57, 8.83)	0.248	1.54 (0.46, 5.19)	0.488
FEV_1_/FVC < LLN	1.11 (0.41, 3.00)	0.840	0.86 (0.30, 2.45)	0.778	1.08 (0.55, 2.12)	0.818	2.15 (0.17, 27.33)	0.554	0.41 (0.20, 5.12)	0.557
**Women**										
**Breathlessness**										
mMRC scale ≥ Grade 2	3.90 (2.00, 7.79)	<0.001	1.37 (0.74, 2.53)	0.313	1.80 (1.19, 2.74)	0.005	3.13 (1.17, 8.32)	0.022	0.95 (0.36, 2.56)	0.924
**Wheeze/whistling**										
Ever	4.62 (2.71, 7.87)	<0.001	1.73 (1.13, 2.66)	0.012	1.73 (1.25, 2.39)	0.001	1.38 (0.57, 3.37)	0.478	2.36 (0.95, 5.81)	0.063
On most days and nights	3.55 (2.06, 6.13)	<0.001	1.53 (0.93, 2.51)	0.095	1.58 (1.10, 2.27)	0.014	1.41 (0.52, 3.80)	0.501	3.31 (1.01, 10.80)	0.048
**Cough/phlegm**										
Chronic cough	0.41 (0.15, 1.18)	0.098	0.41 (0.18, 0.92)	0.031	0.48 (0.26, 0.91)	0.024	0.56 (0.13, 2.46)	0.442	0.10 (0.01, 0.65)	0.016
Chronic phlegm	0.42 (0.15, 1.20)	0.106	0.81 (0.35, 1.86)	0.612	0.62 (0.31, 1.22)	0.165	1.63 (0.31, 8.60)	0.562	0.23 (0.06, 0.95)	0.042
Chronic bronchitis	0.30 (0.08, 1.11)	0.071	0.39 (0.12, 1.30)	0.125	0.35 (0.14, 0.85)	0.021	0.51 (0.06, 4.45)	0.541	0.04 (0.003, 0.58)	0.018
**Airflow obstruction**										
FEV_1_/FVC < 0.70	1.30 (0.67, 2.54)	0.436	0.94 (0.53, 1.67)	0.844	0.90 (0.57, 1.41)	0.647	1.54 (0.54, 4.36)	0.419	2.44 (0.70, 8.44)	0.160
FEV_1_/FVC < LLN	1.67 (0.66, 4.23)	0.281	1.26 (0.62, 2.58)	0.527	1.45 (0.77, 2.74)	0.254	0.88 (0.19, 4.06)	0.873	2.00 (0.29, 13.69)	0.479

*Adjustments for age, sex, educational level, income, BMI, smoking status.

^†^OR for 10-fold increase in pollutant level.

There was an inverse association between FEV_1_ and dyspnoea, ever wheeze and chest illness in the last 12 months in women and with chronic phlegm in men after adjusting for height, age, education, BMI, income and smoking status (Additional file 3: Table S2).

## Discussion

This study shows that the risk of reporting wheeze (ever and on most days and nights) and dyspnoea (mMRC scale ≥2) were significantly higher among those exposed to biomass smoke, particularly in women. These respiratory symptoms were also positively associated with quantitative measures of PM_2.5_ greater than two standard deviation of the mean but not to the 24-h average mean, suggesting that peaks of pollution may be more important than average exposures. Those exposed to biomass smoke who had respiratory symptoms were more likely to have lower lung function and the prevalence of airflow obstruction was significantly higher amongst the older individuals.

All respiratory symptoms were self-reported without further clinical assessment, which may have resulted in misclassification. People in low-income countries often consider wheeze, breathlessness and bringing up phlegm to some extent as normal which may result in under-reporting of symptoms and if this was differentially expressed between exposed and non-exposed groups this may underestimate the true risk. The respiratory questionnaire used was developed and validated in developed countries and although our version was back translated to ensure best delivery of questions interpretative issues may have arisen. For instance, there is no terminology in Nepali for the term “wheeze” which could have caused some confusion among interviewees but efforts were made to minimise these by using bilingual speakers from their local communities trained in questionnaire delivery.

The major strengths of this study are its size, the use of a comparator group (studying biomass smoke exposed and non-exposed groups) and the adjustment for confounders, often inadequately dealt with in previous studies. We included young adults (≥16 years) because in Nepal cooking is usually delegated to adolescents, particularly girls and those living in the rural areas.

Previous work has studied populations using different types of biomass smoke for varying lengths of time making comparisons difficult especially when the issues of confounding is inconsistently addressed. Behera and Jindal [11] reported prevalences of respiratory symptoms for different types of fuel users (biomass, kerosene, LPG and mixed) in Indian women and found that respiratory symptoms did not follow any clear pattern: chronic bronchitis was greater in biomass users, cough greater in kerosene users and breathlessness in mixed fuel users. Other studies [12, 13] have not found an association between exposures to wood smoke and respiratory symptoms but a randomised controlled trial study in Guatemala [14] of respiratory symptoms in women involved in cooking reported that women provided with improved cook stoves reported significantly less wheeze compared to baseline but with no significant reduction in other respiratory symptoms.

Our study recorded significantly higher “ever wheeze” in the rural compared to the urban area, similar to previous reports from Nepal (wood smoke) [6, 15], Canada [16, 17] (smoke from burning agricultural residue and wood), India [11] (biomass smoke), China [18, 19] (coal smoke) and Guatemala [20] (wood smoke). The presence of significantly higher prevalence of wheeze (ever, on most days/nights, and in the last 12 months) in the rural, life-long non-smoking population further suggests that the risk of being wheezy is likely to increase in populations exposed to biomass smoke. The risk of ever wheeze in biomass smoke exposed women was 60% higher compared to men but there were no significant differences between urban males and females. The risk of ever wheeziness increased with age for the biomass exposed population but not for the non-exposed population suggesting that prolonged exposure to biomass smoke increases the risk although in other studies respiratory symptoms have generally increased with age. Ex-smokers showed more than a six-fold increase in ever wheeze in the biomass smoke exposed population and a three-fold increase in the urban population compared to life-long non-smokers whereas the additional risks in current smokers were nearly three and two fold respectively. The higher risk in the ex-smokers could be explained as the ex-smokers gave up smoking only after they were medically diagnosed with respiratory problems which might have persuaded them to quit smoking. Similar results for ex- and current smokers were found for wheeze in the last 12 months.

Self-reported chronic phlegm was significantly higher in non-smoking, non-biomass exposed men (6.7%) and women (4.9%) compared to the exposed group (men 2.7%, women 2.0%), contrary to the findings reported by other studies in Nepal [6] and other countries [11, 19]. This finding is both marked and surprising and while this might be due to bias in reporting, the risk in the urban population might be real. Most of the urban dwellers were exposed to biomass at some point in their early years and there might have been a residual effect of that early pollutant exposure but this should not overwhelm current exposure. This finding could also possibly be due to the higher urban outdoor air pollution concentrations from vehicle generated pollutants although other causes unrelated to air quality such as post nasal discharge may be a possibility. There have been abundant studies in industrialised countries on the short and long term health effects of vehicle generated ambient air pollution, with special emphasis on respiratory and cardiovascular health effects [21–23] showing positive correlations between these health outcomes and concentrations of ambient air pollutants. It is possible that vehicle generated particles released in ambient air are more toxic in the context of mucus hyper-secretion compared to biomass smoke and thus cause more respiratory symptoms but future studies are needed to assess the differential toxicity due to different types of fuel [24, 25] and also compare with existing toxicity data from vehicle generated particles.

Unsurprisingly production of cough and phlegm was more common in both current and ex-smokers and also in older age groups. Phlegm production was greatest in the biomass exposed illiterate population indicating that socio-economic status is a risk factor and living within a kitchen with no ventilation increased phlegm production in both the rural and urban populations but not statistically significantly so. This indicates that exposure to kitchen fumes (smoke from fuel burning and also mist from cooking oil when heated) might be a risk factor, again a potential surrogate indicator of exposure.

Biomass smoke exposed men and women reported more breathlessness compared to their non-exposed counterparts but the difference was only significant for biomass smoked compared to non-biomass exposed males. Recorded breathlessness in this study is lower compared to other published studies. As the biomass smoke exposed area was in a hilly region, the population might have attributed breathlessness to exertion rather than to inhalation of biomass smoke, especially as females do a lot of manual work in the fields. It is also possible that our sample is relatively young (mean age 35 years), hence less likely to have (and admit to have) dyspnoea. Cooking with kerosene increased the risk of developing breathlessness in urban dwellers (results not shown) but this result was based on a very small number of individuals and could just be a chance finding.

Direct measurement of exposure as mean 24-h indoor PM_2.5_ in this study failed to show any significant associations with respiratory symptoms but some of the proxy measurements such as use of biomass, illiteracy and poor ventilation (data not shown) showed a relationship suggesting that exposure to biomass smoke might be a risk for respiratory symptoms. Of interest is that when we considered peak smoke exposures (taken as mean PM_2.5_ >2 SDs above the 24-h mean) a relationship between respiratory symptoms and exposures emerged, suggesting that time spent at concentrations that are considerably higher than background may be more important than consistently high exposures.

The odds of presence of airflow obstruction was greater amongst those exposed to biomass although it did not reach statistical significance. The higher prevalence of respiratory symptoms such as wheeze and breathlessness without having airflow obstruction could be due to the high proportion of younger individuals in our sample. Alternatively, it is possible that spirometry was not sensitive enough to detect the very early stage of airflow obstruction.

## Conclusions

In summary, in this study the prevalence of wheeze in a biomass smoke exposed population is in line with most of the previous findings in Nepal and other low-income countries but the results for cough differ. This ambiguity regarding the cough and phlegm results from urban Nepal should be interpreted with special attention. Future studies in urban Nepal looking at respiratory symptoms should be looking at all the aspects like toxicity of outdoor pollutants and whether any other risk factors are confounding the results.

## Electronic supplementary material

Additional file 1: Figure S1: Typical temporal profiles of PM_2.5_ and CO concentrations. (DOCX 5 MB)

Additional file 2: Table S1: Respiratory symptoms in Nepalese adult men and women according to household fuel type, stratifying for age in tertiles. (DOCX 36 KB)

Additional file 3: Table S2: Regression coefficients of lung function indices using robust variance estimates. (DOCX 38 KB)

## Abbreviations

BMI: Body mass index
CI: Confidence interval
CO: Carbon monoxide
COPD: Chronic obstructive pulmonary disease
ECRHS: European community respiratory health survey
FEV1: Forced expiratory volume in 1 second
FVC: Forced vital capacity
LLN: Lower limit of normal
LPG: Liquefied petroleum gas
MRC: Medical Research Council
OR: Odds ratio
PM: Particulate matter
SD: Standard deviation
VDC: Village development community.
