# Supplementary material for: The effect of exposure to biomass smoke on respiratory symptoms in adult rural and urban Nepalese populations
Source: Environ Health. 2014 Nov 6;13:92. doi: 10.1186/1476-069X-13-92 (PMC4232609; doi:10.1186/1476-069X-13-92)
Supplement: Supplementary file 1 — Additional file 1: Figure S1: Typical temporal profiles of PM2.5 and CO concentrations. (DOCX 5 MB) [file 12940_2014_794_MOESM1_ESM.docx]

**
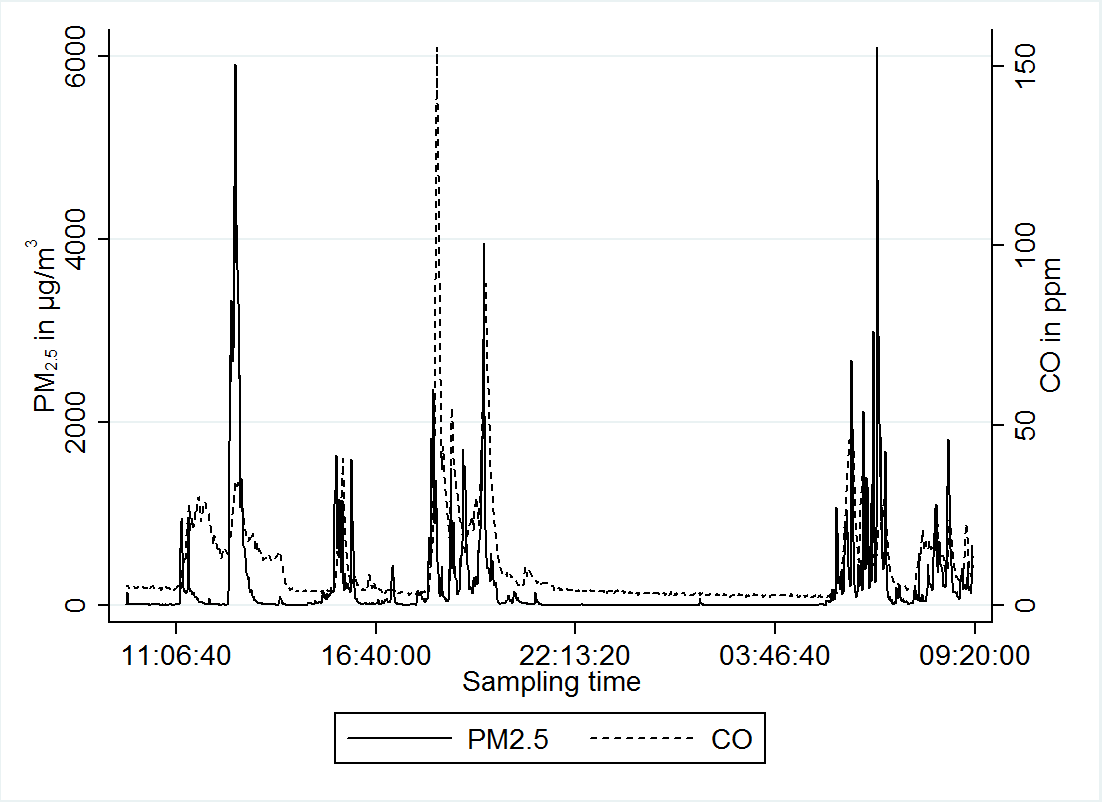

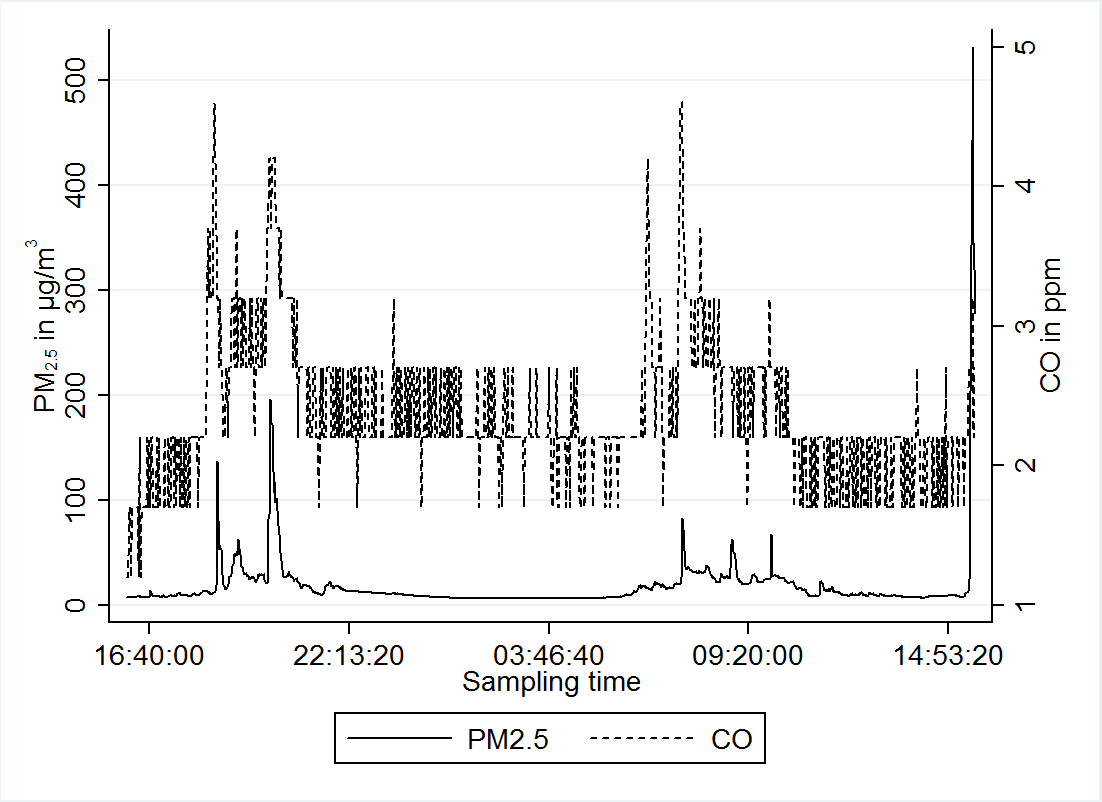
**

**Figure S1**: Typical temporal profiles of PM_2.5_ and CO concentration (Nepalese data). (a) Topl: Wood burnt in a 3-stone stove. Afternoon snacks prepared during 1400-1445, evening meal prepared during 1900-2000 and morning lung prepared during 0700-0830 hours and (b) Bottom: LPG fuel burnt in gas stove. Afternoon snacks prepared during 1515-1530, evening meal prepared during 1800-1900, morning breakfast prepared during 0445-0600 and morning lunch prepared during 0700-0800.
