# Supplementary material for: The effect of exposure to biomass smoke on respiratory symptoms in adult rural and urban Nepalese populations
Source: Environ Health. 2014 Nov 6;13:92. doi: 10.1186/1476-069X-13-92 (PMC4232609; doi:10.1186/1476-069X-13-92)
Supplement: Supplementary file 2 — Additional file 2: Table S1: Respiratory symptoms in Nepalese adult men and women according to household fuel type, stratifying for age in tertiles. (DOCX 36 KB) [file 12940_2014_794_MOESM2_ESM.docx]

Table S1: Respiratory symptoms* in Nepalese adult men and women according to household fuel type, stratifying for age in tertiles

|  |  | **Men** | | | | |  | **Women** | | | | |
| --- | --- | --- | --- | --- | --- | --- | --- | --- | --- | --- | --- | --- |
|  |  | **Biomass** | | **Non-biomass** | |  |  | **Biomass** | | **Non-biomass** | |  |
|  |  | **n** | **% (95% CI)** | **n** | **% (95% CI)** | **p** |  | **n** | **% (95% CI)** | **n** | **% (95% CI)** | **p** |
| 1^st^ tertile (16-25 years) |  | 139 |  | 127 |  |  |  | 163 |  | 158 |  |  |
| mMRC scale ≥ Grade 2 |  | 9 | 6.5 (2.4, 10.6) | 0 | - | - |  | 11 | 6.6 (2.9, 10.3) | 3 | 1.9 (-0.3, 4.1) | 0.035 |
| Wheeze |  |  |  |  |  |  |  |  |  |  |  |  |
| Ever |  | 19 | 13.3 (7.8, 18.8) | 8 | 6.5 (1.7, 11.3) | 0.071 |  | 24 | 14.9 (9.3, 20.6) | 12 | 7.5 (3.1, 11.9) | 0.042 |
| On most days/nights |  | 13 | 9.1 (45, 13.8) | 5 | 4.0 (-0.1, 8.1) | 0.115 |  | 20 | 12.3 (7.1, 17.4) | 9 | 5.7 (2.1, 9.3) | 0.040 |
| Chronic cough |  | 3 | 1.9 (-0.2, 3.9) | 1 | 0.9 (-0.8, 2.7) | 0.493 |  | 2 | 1.3 (-1.2, 3.8) | 3 | 1.8 (-0.1, 3.7) | 0.753 |
| Chronic phlegm |  | 1 | 0.7 (-0.7, 2.1) | 4 | 3.1 (0.2, 6.0) | 0.140 |  | 3 | 2.0 (-0.2, 4.1) | 11 | 6.6 (3.0, 10.1) | 0.029 |
| Chronic cough and phlegm |  | 1 | 0.7 (-0.7, 2.1) | 0 | - | - |  | 1 | 0.6 (-0.6, 1.9) | 3 | 1.8 (-0.1, 3.7) | 0.293 |
|  |  |  |  |  |  |  |  |  |  |  |  |  |
| 2^nd^ tertile (26-40 years) |  | 119 |  | 125 |  |  |  | 151 |  | 137 |  |  |
| mMRC scale ≥ Grade 2 |  | 9 | 7.6 (2.8, 12.4) | 0 | - | - |  | 25 | 16.4 (10.5, 22.3) | 6 | 4.4 (1.0, 7.9) | <0.001 |
| Wheeze |  |  |  |  |  |  |  |  |  |  |  |  |
| Ever |  | 19 | 15.8 (9.2, 22.3) | 9 | 7.3 (2.9, 11.8) | 0.037 |  | 44 | 28.9 (21.6, 36.2) | 6 | 4.4 (1.0, 7.9) | <0.001 |
| On most days/nights |  | 15 | 12.4 (6.5, 18.2) | 7 | 5.7 (1.7, 9.8), | 0.066 |  | 37 | 24.5 (17.6, 31.3) | 5 | 3.7 (0.5, 6.8) | <0.001 |
| Chronic cough |  | 4 | 3.3 (0.1, 6.6) | 5 | 4.0 (0.6, 7.4) | 0.783 |  | 5 | 3.3 (0.4, 6.1) | 3 | 2.2 (-0.3, 4.7) | 0.599 |
| Chronic phlegm |  | 2 | 1.7 (-0.1, 4.0) | 20 | 16.1 (1.0, 22.3) | <0.001 |  | 5 | 3.3 (0.5, 6.2) | 6 | 4.3 (0.9, 7.8) | 0.664 |
| Chronic cough and phlegm |  | 2 | 1.7 (-0.6, 4.0) | 5 | 4.0 (0.6, 7.4) | 0.272 |  | 3 | 2.0 (-0.3, 4.3) | 2 | 1.5 (-0.6, 3.5) | 0.734 |
|  |  |  |  |  |  |  |  |  |  |  |  |  |
| 3^rd^ tertile (≥41 years) |  | 124 |  | 128 |  |  |  | 149 |  | 128 |  |  |
| mMRC scale ≥ Grade 2 |  | 30 | 21.5 (1.9, 28.1) | 9 | 8.2 (3.3, 13.1) | 0.002 |  | 50 | 32.1 (24.7, 39.6) | 21 | 17.7 (11.0, 24.4) | 0.005 |
| Wheeze |  |  |  |  |  |  |  |  |  |  |  |  |
| Ever |  | 53 | 40.6 (32.3, 49.0) | 15 | 00.7 (6.7, 18.7) | <0.001 |  | 84 | 55.0 (47.1, 62.8) | 23 | 19.4 (12.3, 26.5) | <0.001 |
| On most days/nights |  | 43 | 33.3 (25.2, 41.5) | 10 | 8.3 (3.4, 13.2) | <0.001 |  | 65 | 42.4 (34.5, 50.4) | 16 | 13.3 (7.4, 19.2) | <0.001 |
| Chronic cough |  | 12 | 8.5 (4.0, 12.9) | 13 | 11.7 (5.9, 17.5) | 0.395 |  | 15 | 9.1 (4.7, 13.5) | 10 | 8.9 (3.8, 14.0) | 0.952 |
| Chronic phlegm |  | 9 | 7.0 (2.5, 11.4) | 24 | 19.4 (12.3, 26.4) | 0.004 |  | 11 | 6.7 (2.9, 10.5) | 7 | 6.2 (1.8, 10.6) | 0.861 |
| Chronic cough and phlegm |  | 6 | 4.4 (0.9, 7.9) | 8 | 6.9 (2.2, 11.5) | 0.411 |  | 9 | 5.4 (1.9, 8.9) | 4 | 3.7 (0.1, 7.2) | 0.495 |

*Adjusted for age
