# Supplementary material for: The effect of exposure to biomass smoke on respiratory symptoms in adult rural and urban Nepalese populations
Source: Environ Health. 2014 Nov 6;13:92. doi: 10.1186/1476-069X-13-92 (PMC4232609; doi:10.1186/1476-069X-13-92)
Supplement: Supplementary file 3 — Additional file 3: Table S2: Regression coefficients of lung function indices using robust variance estimates. (DOCX 38 KB) [file 12940_2014_794_MOESM3_ESM.docx]

Table S2: Regression coefficients of lung function indices using robust variance estimates

| **Respiratory symptoms** | **FEV_1_** | | | **FVC** | | **FEF_25-75_** | | **FEV_1_/FVC** | |
| --- | --- | --- | --- | --- | --- | --- | --- | --- | --- |
|  | **β (95% CI)** | | **p** | **β (95% CI)** | **p** | **β (95% CI)** | **p** | **β (95% CI)** | **p** |
| **Men** | | | | | | | | | |
| **Breathlessness** |  |  | |  |  |  |  |  |  |
| mMRC grade ≥2 | -0.243 (-0.440, -0.046) | 0.016 | | -0.284 (-0.494, 0.075) | 0.008 | -0.094 (-0.442, 0.256) | 0.599 | -2.838 (-6.683, 1.007) | 0.148 |
| **Wheeze** |  |  | |  |  |  |  |  |  |
| Ever | -0.276 (-0.407, -0.144) | <0.001 | | -0.220 (-0.357, -0.084) | 0.002 | -0.310 (-0.542, -0.077) | 0.009 | -4.972 (-7.332, -2.611) | <0.001 |
| On most days and nights | -0.375 (-0.541, -0.209) | <0.001 | | -0.302 (-0.469, -0.135) | <0.001 | -0.447 (-0.719, -0.176) | 0.001 | -6.262 (-9.229, -3.295) | <0.001 |
| **Cough/phlegm** |  |  | |  |  |  |  |  |  |
| Chronic cough | -0.117 (-0.361, 0.128) | 0.348 | | -0.176 (-0.457, 0.105) | 0.219 | 0.019 (-0.299, 0.337) | 0.905 | -0.569 (-4.451, 3.313) | 0.774 |
| Chronic phlegm | -0.205 (-0.386, -0.023) | 0.027 | | -0.217 (-0.409, -0.026) | 0.026 | -0.068 (-0.364, 0.228) | 0.652 | -2.435 (-5.327, 0.457) | 0.099 |
| Chronic bronchitis | -0.283 (-0.578, 0.011) | 0.059 | | -0.363 (-0.713, -0.013) | 0.042 | -0.116 (-0.475, 0.242) | 0.524 | -2.022 (-6.756, 2.712) | 0.402 |
|  |  |  | |  |  |  |  |  |  |
| **Women** | | | | | | | | | |
| **Breathlessness** |  |  | |  |  |  |  |  |  |
| mMRC grade ≥2 | -0.102 (-0.191, -0.013) | 0.024 | | -0.121 (-0.221, -0.020) | 0.018 | -0.090 (-0.286, 0.106) | 0.368 | -1.636 (-4.228, 0.956) | 0.215 |
| **Wheeze** |  |  | |  |  |  |  |  |  |
| Ever | -0.108 (-0.184, -0.032) | 0.006 | | -0.068 (-0.157, 0.020) | 0.127 | -0.174 (-0.322, -0.026) | 0.021 | -2.858 (-4.788, -0.929) | 0.004 |
| On most days and nights | -0.077 (-0.160, 0.006) | 0.069 | | -0.017 (-0.114, 0.080) | 0.726 | -0.154 (-0.317, 0.009) | 0.064 | -3.378 (-5.602, -1.154) | 0.003 |
| **Cough/phlegm** |  |  | |  |  |  |  |  |  |
| Chronic cough | -0.125 (-0.266, 0.017) | 0.085 | | -0.195 (-0.345, -0.044) | 0.011 | 0.046 (-0.249, 0.341) | 0.759 | -0.242 (-5.320, 4.835) | 0.925 |
| Chronic phlegm | -0.045 (-0.167, 0.076) | 0.463 | | -0.068 (-0.204, 0.069) | 0.330 | -0.007 (-0.257, 0.243) | 0.958 | -1.080 (-4.533, 2.372) | 0.539 |
| Chronic bronchitis | -0.054 (-0.234, 0.127) | 0.559 | | -0.171 (-0.371, 0.301) | 0.096 | 0.124 (-0.262, 0.511) | 0.528 | 1.672 (-2.931, 6.276) | 0.476 |

* Adjusted for age, education, height, BMI, income, smoking history
